# Swiss National Research Programme “Opportunities and Risks of Nanomaterials” (NRP 64): key findings

**DOI:** 10.1186/s12951-017-0282-5

**Published:** 2017-06-24

**Authors:** Ueli Aebi, Peter Gehr

**Affiliations:** 1 Steering Committee of the Swiss National Research Programme NRP 64 and Prof. Emeritus, Biozentrum University of Basel, Basel, Switzerland; 2 Steering Committee of the Swiss National Research Programme NRP 64 and Prof. Emeritus, University of Bern, Bern, Switzerland

This *Special Issue* is devoted to the outcome of a 5-year Swiss *National Research Programme* on the “Opportunities and Risks of Nanomaterials” (NRP 64). Nanotechnology is an enabling technology that explores the structure and function of naturally occurring nanomaterials, as a basis for engineering synthetic nanomaterials inspired by nature. Nanomaterials have rapidly come to play an important role in healthcare, the consumer industry, energy storage and other areas. NRP 64 comprised 23 mostly interdisciplinary research projects that studied the impact of engineered nanomaterials on the environment and on human health. The outcome of the programme has been highly satisfying, as all projects yielded new and in some cases unexpected results [1]. In this special issue, we present the key findings of 15 projects, some in the form of reviews rather than original research articles to put things into a broader perspective.

As the title of NRP 64 “Opportunities and Risks of Nanomaterials” implies, the programme’s primary goal was to investigate potential applications of existing nanomaterials in healthcare, the consumer industry and in the environment, and to identify, characterise and minimise the possible risks associated with their use. Several projects dealt with the development and/or testing of tools and measurement protocols to track the fate, effect or biodegradation of nanoparticles in cells, tissues, soil and aquatic environments. Highlights of the programme included: single cell surgery by metal nanomagnets; biomedical nanoparticles as immune modulators [2]; novel nanoparticles for efficient and safe drug delivery; nanofiber-reinforced bone substitute materials; aerogels and new tissue engineering scaffolds (e.g. artificial cartilage) made of cellulose nanocrystals; nanoparticle transport across the human placenta; transport of nanoparticles after release from biodegradable implants; non-invasive monitoring of the interaction between nanoparticles and aquatic microorganisms [3]; evaluation platforms for safety and environmental risks of carbon nanotube reinforced nanocomposites [4]; development of a “lab-on-a-chip” tool to rapidly assess the safety of novel nanoscale active materials for next-generation battery systems.

NRP 64 has undeniably generated a large amount of new knowledge about the use, application and risk assessment of nanomaterials. This will enable Switzerland to remain at the cutting edge of efforts to develop smart and novel nanomaterials that are inspired by nature, and to look for new applications that minimise their health and environmental risks. In addition, when it comes to a more physiological or pathological understanding of how nanomaterials interact with cells, tissues and the environment, we have definitely made significant progress. What is still lacking are long-term studies that clearly document how exposure to nanoparticles over a longer period affects our bodies and the environment. By the same token, we still know very little about the effects of indirect exposure to nanoparticles, e.g. through the accumulation of nanoparticles in plants or animals that will eventually end up in our food chain.

One of the general conclusions drawn from NRP 64 is the following: whenever a new nanomaterial is identified or a new application of an established nanomaterial pursued, carrying out a risk re-assessment is essential to guarantee safety. Overall, the programme has clearly shown that, for the nanomaterials investigated, the opportunities outweigh the risks. Not only has NRP 64 taken the research field a big step forward, its results have made it evident where more basic research and/or a more detailed risk assessment are necessary before researchers can start exploiting the application potential of individual nanomaterials.
